# Quality assessment of antibiotic use data in the Norwegian veterinary prescription register for 2023

**DOI:** 10.1186/s12917-025-04903-9

**Published:** 2025-08-07

**Authors:** Trishang Udhwani, Kari Grave, Petter Hopp, Kari Olli Helgesen

**Affiliations:** Norwegian Veterinary Institute, Elizabeth Stephansens vei 1, Ås, 1433 Norway

**Keywords:** Antibiotic use data, Data quality, Data validation, Animal health data, Secondary data, Data accuracy, Data completeness, Data timeliness

## Abstract

**Background:**

Antibiotic use data in animals is of interest to understand the development and occurrence of antibiotic resistance from a One Health perspective. Since 2023, it has been mandatory to collect and report data on antibiotic use per animal species or subcategory of species for cattle, pigs, chickens and turkeys in all EU and EEA countries. Norway collects data on use of medicines for animals through the Norwegian Veterinary Prescription Register (VetReg). It is mandatory for veterinarians to report the use of all medicines for food-producing animals and for pharmacies to report to VetReg all medicines dispensed to animal owners for all animal species and to veterinarians. The aim of our study was to evaluate the quality of antibiotic VetReg data for 2023, focusing on the information required to fulfill reporting of use data in accordance with the EU-requirements. The quality attributes, accuracy of data and timeliness of reporting, were evaluated using VetReg data, and completeness was evaluated by comparing VetReg data with sales statistics from wholesalers.

**Results:**

In general, accuracy varied between the variables and within a variable, depending on whether veterinarians or pharmacies were the data source. For example, 97% of veterinary records included the required subcategory of the animal species, while only 17% of the pharmacy’s records did. Antibiotic use data in VetReg were calculated to cover 85% of the antibiotics sold by wholesaler to pharmacies. Timeliness also varied - i.e. pharmacies reported almost immediately after dispensing, while 29% of veterinary records were not reported within the deadline of seven days.

**Conclusions:**

Antibiotic use data for animals in Norway in 2023 could be reported to EMA using VetReg data. The data were, however, not fully accurate and complete. This study revealed several specific accuracy issues and issues with timeliness of reporting. These findings provide the basis for targeted quality improvement measures and can be transformed to metrics suitable to track the progress of the ongoing quality improvement work.

**Supplementary Information:**

The online version contains supplementary material available at 10.1186/s12917-025-04903-9.

## Background

Antibiotic resistance is an emerging threat to humans but also animal health. Use of antibiotics selects for antibiotic resistance and knowledge of the use and the use patterns is necessary to understand the occurrence and development of resistance [1, 2]. Reliable antibiotic sales and use (ASU) data are needed to evaluate e.g. disease prevention measures, antimicrobial stewardship efforts and reduction targets for antibiotic use set as part of sector-wise, national or regional strategies. In addition, reliable data are essential if use data of antibiotics are to be compared, such for benchmarking.

Several integrated analyses have shown positive associations between the use of certain antibiotic classes/sub-classes in food-producing animals and the occurrence of resistance to these in clinical isolates of bacteria obtained from humans [3]. Use of antibiotics in animals can also cause a spill-over of antibiotics to the surrounding environment through discharge of medicated feed and medicated drinking-water, and as residuals in excrements used as fertilizers [4]. Antibiotic use data for animals is therefore of interest from a One Health perspective. In addition, antibiotic resistance can challenge the effect of treatment of bacterial diseases in animals [5]. With regards to food-producing animals, there are also food-safety concerns with antibiotic residues in the end-products [6]. To elucidate the relationship between use and the risk of these unwanted consequences of antibiotic use in animals, it is vital to have access to accurate data on use.

Sales data for veterinary antibiotics have been voluntarily submitted to the European Medicines Agency (EMA) by European Union (EU) and European Economic Area (EEA) countries yearly from 2010 to 2022 through the European Surveillance of Veterinary Antimicrobial Consumption (ESVAC) [7].

In 2019, a new EU-regulation on veterinary medicinal products was passed (EU 2019/6) that requires EU Member States to collect relevant and comparable data on the volume of sales of antimicrobial veterinary medicinal products (VMPs) and on the use of antimicrobial medicinal products [both VMPs and Human Medicinal Products (HMPs)], i.e. antibiotics, antivirals, antifungals and anti-protozoals, in animals [8]. Through the delegating regulation (EU 2021/578) the antimicrobials have been split into those mandatory and those voluntary to be reported, of which the mandatory sales and use data to be reported represent substances with effect against bacteria (hereafter referred to as antibiotics) [9]. The implementing regulation (EU 2022/209) establishes the format for reporting of the sales and use data [10].

According to the regulation, the sales and use data shall be “accurate, complete and consistent”.

A six-year stepwise approach is set for the reporting [9]. The first step is mandatory annual reporting of antimicrobial use (AMU) in cattle, swine, broilers and turkeys (for two to four categories per species) from 2023 onwards. These species have been prioritized because they are the major food-producing species in EU/EEA countries overall and antibiotic resistance data are collected from the same species and categories of these at an EU-level, thus allowing for e.g. integrated analysis [3]. The next step is reporting of AMU for all food-producing species and the last step will require reporting of AMU also for cats, dogs and fur-animals.

The delegating regulation (EU 2021/578) further requires that all countries “set out a data quality management plan that comprises appropriate data quality management procedures, including procedures for data quality assurance, validation and quality control” [9].

EMA’s report with results from the first year of ASU data collection was published in 2025. Sales data was presented approximately as in previous ESVAC reports, but no quantitative analysis of the use data were made. The latter was explained with various use data quality, especially with regards to coverage, that lead to data not representing actual use [11].

Norway has collected antibiotic sales data for farmed fish since 1981 and for terrestrial animals since 1993 and have reported sales data to EMA on a voluntary basis since the start of the ESVAC project. Through its membership of EEA Norway is obliged to implement EU 2019/6 including the related delegating regulations [7, 8, 12, 13].

Data on use for all veterinary medicinal products (VMP) and human medicinal products (HMP), have been collected in Norway since 2011 for farmed fish and from 2012 for all food-producing species, including all use for horses [14]. Use data are collected by the Norwegian Food Safety Authority (NFSA) through the Veterinary Prescription Register (VetReg). Data sources are veterinarians, pharmacies and feed mills (for farmed fish).

VetReg was established prior to the delegating regulation (EU 2021/578). To meet with the requirements in this regulation, the only adjustment to the system before 2023 was to implement reporting by categories of cattle, pigs, chickens and turkeys [15]. The quality of antibiotic use data for terrestrial animals in VetReg, for the years 2015–2018, have previously been investigated. For some pharmaceutical forms, especially for intramammaries, which are antibiotics inserted into the mammary ducts for local treatment of infections of the udder, and oral pastes, the quality of data reported by veterinarians was sub-optimal [16–18]. This had partly to do with the fact that veterinarians can select a variety of units for the amounts of the VMP or HMP administered to the animals or delivered to the animal owner when reporting the data to VetReg. This makes it difficult to standardise calculation of amounts reported used per active antibiotic substance per record. Recently, the quality of fish vaccine data in VetReg was analyzed and issues were identified with different variables such as number of animals, weight, and species [19].

To fulfill the requirements set in EU regulation 2021/578, the quality of VetReg data needed to be assessed, i.e. to perform “data quality assurance, validation and quality control”. Through such work, quality issues and metrics useful for tracking the progress of improving the quality can be identified.

The main aim of this study was to evaluate the quality of VetReg antibiotic use data for 2023, focusing on the data that will be used to fulfill the requirements of the first step of use data mandatory to be reported to EMA. This includes developing and describing the methodology for the data cleansing steps performed. The study followed the framework suggested by Birkegård et al. (2018) for data quality evaluation of national animal health registers [20]. It includes a description of the data in VetReg and the other data sources used for evaluation of VetReg, the use of relevant quality attributes as well as the identification of quality issues.

## Methods

### Data sources

**Fig. 1:**
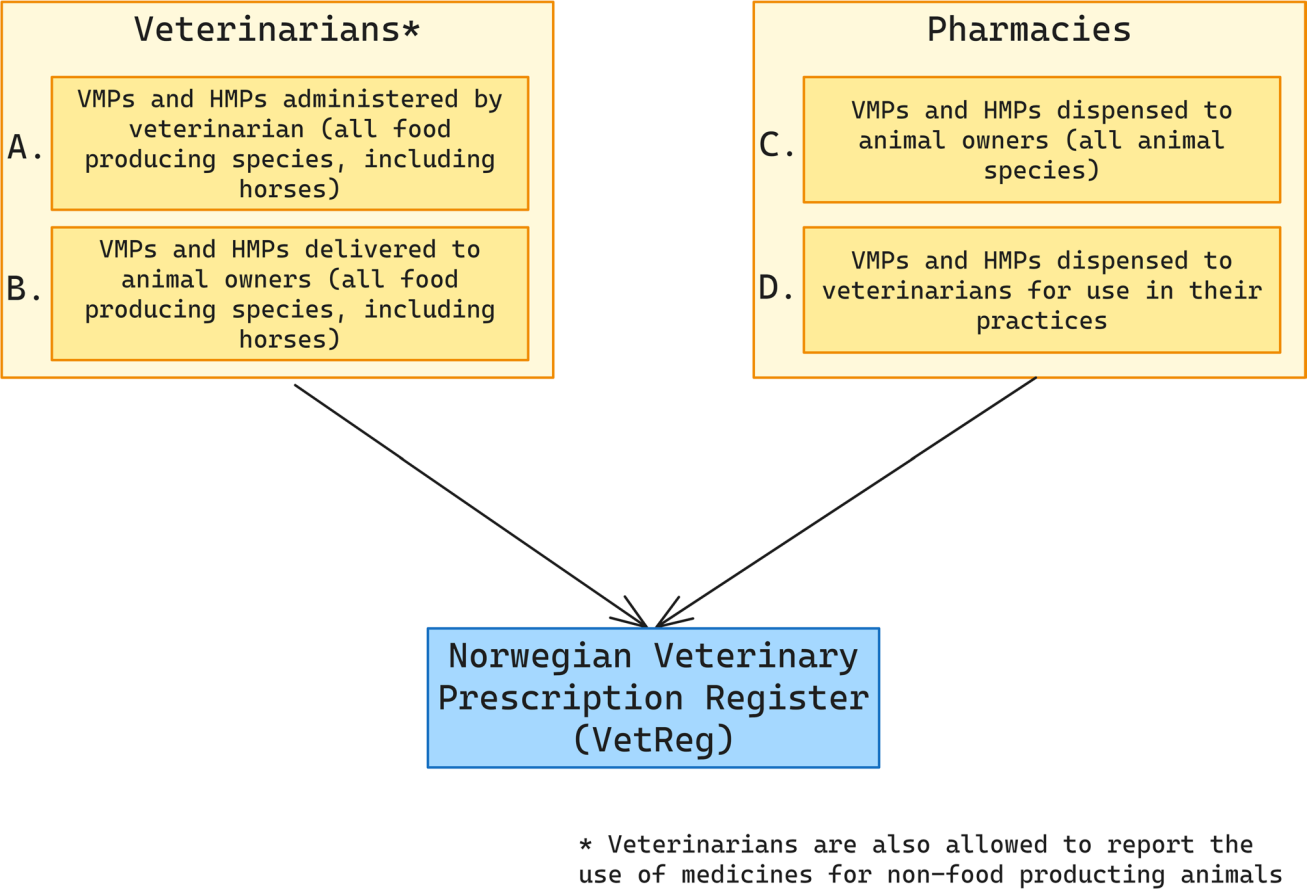
Reporting of data to VetReg as of 2023. Figure showing who reports data to VetReg and what type of data is mandatory to report. **A** and **B** are the two types of data mandatory to report by veterinarians and **C** and **D** are the two types of data mandatory to report by pharmacies. VMPs: Veterinary Medicinal Products, HMPs: Human Medicinal Products

Data on the use of medicinal products in animals has been collected by NFSA through VetReg and the type of data reported to VetReg are shown in Fig. 1. It is mandatory for pharmacies to report all medicines dispensed to animal owners (i.e. for all animal species) and to veterinarians for use in their practice. Veterinarians must report all their use and administration of medicines to food-producing animals (including horses), while it is voluntary to report use for other species kept or bred e.g. companion animals and fur animals. Veterinarians are allowed to use or prescribe medicines for other species and other indications than these are authorised for, including HMPs, however, such use has to be in accordance with Article 112–114 of EU regulation 2019/6 [8].

Data to VetReg are submitted via pharmacies’ accounting systems and veterinarians’ practice management software programs, respectively, and via system-to-system communication with VetReg. The data can also be reported to VetReg using NFSA’s web portal. NFSA accepts corrections in VetReg within six months of the original reporting.

In the following, one line in VetReg is referred to as one record; this applies both for records from pharmacies and from veterinarians. One record contains information about the use or dispensing of one VMP or HMP on one occasion to one animal or a group of animals. Records representing veterinarians’ use and pharmacies dispensing to animal owners, respectively, should provide the species of the treated animal, and since 2023 additionally provide the animal category for cattle, pigs, chickens and turkeys, e.g. differentiate between dairy cattle and beef cattle. All variables in a record are shown in Supplementary Table 1.

For pharmacies, the system is designed for reporting the amount of the VMP and HMP dispensed to the animal owners and veterinarians, almost exclusively as number of packages (and part of packages when relevant). The VetReg variable name is “number_of_packages” (Table 1). Veterinarians report the amount in e.g. mL, g or dose of the VMP or HMP. The VetReg variables are “dispensed_quantity” and “dispensed_unit” (Table 1).

For veterinarians, the use must be reported to VetReg within seven days, while no reporting deadline has been established for prescriptions dispensed by pharmacies, to the best of our knowledge [21]. The reason behind this specific discrimination between veterinarians and pharmacies is not known to us. Access to VetReg is restricted but is made available for the Norwegian Veterinary institute (NVI) by NFSA through a mutual agreement. The first VetReg data for 2023 were made available for NVI on 11.01.2024 and VetReg-data for NVI was thereafter updated daily. For this study, the data were downloaded as csv-files on 15.03.2024, meaning that 2023-data reported to VetReg later than 15.03.2024 were not included in the current study.

The Norwegian Medicinal Products Agency (NoMA) owns and maintains a database, called FEST, containing information about medicinal products. The main purpose of this database is to support prescribing and dispensing of medicines in Norway (in Norwegian: Forskrivnings- og ekspedisjonsstøtte, abbreviated FEST) [22]. The FEST database was used to supplement VetReg data and sales data as referred to in the section “Preparation of data for further analysis” below.

To assess the accuracy and completeness of antibiotic VMP use data reported to VetReg, covering the Anatomical Therapeutic Chemical veterinary (ATCvet) codes for which use is mandatory to be reported to the European Medicines Agency according to the delegating regulation (EU 2021/578) (see Supplementary Table 2), sales data for antibiotic VMPs with the same codes were used. These data were collected from the Norwegian Institute of Public Health (NIPH) as wholesalers are mandated to report their sales of VMPs (and HMPs) to pharmacies directly to NIPH. These data are used for the reporting of sales data to EMA. Sales of HMPs by wholesalers were excluded from this analysis because it is not known if the HMPs sold to pharmacies are used for humans or animals. The variables collected on sales of antibiotic VMPs are, among others, ATCvet code, name of the VMP and the number of packages sold per Nordic article number (common article numbers per VMP and HMP used by all stakeholders) [23].

For the wholesalers, the deadline for the reporting of annual sales data for the preceding year to NIPH is January 15th. NIPH provided NVI with sales data for 2023 on 06.02.2024 for the ATCvet codes shown in Supplementary Table 2, which covers data of antibiotic VMPs mandatory to be reported to EMA [9].

### Preparation of data for further analysis

As a first step, VetReg data were supplemented with FEST data using the Nordic article number as the common identifier. Variables added from FEST are shown in Table 1. Each pharmaceutical form provided in FEST was thereafter assigned to the same groups of pharmaceutical forms as those given in the Antimicrobial Sales and Use (ASU) technical implementation protocol [24]. This grouping decreased the number of pharmaceutical forms.

**Table 1:** List of variables included in the datasets made for quality analysis of VetReg in 2023

VetReg variables	FEST variables	Support registry variables
report_id	nordic_article_number	conversion factor
registered date	active substance(s)	pharmaceutical form
delivery date	strength of active substance	
delivery type	strength unit of active substance	
animal_category	strength denominator of active substance	
animal_id	strength denominator unit of active substance	
number_of_animals	pharmaceutical form expanded	
prescription_reason (short and broard)	package size	
diagnosis	package size unit	
nordic_article_number	ATC code	
report_id_two	medicinal_product_name	
dispensed_quantity		
dispensed_unit		
number_of_packages		
medicinal_product_name		

The list is subdivided by the registries they originate from: VetReg, the national registry supporting prescribing and dispensing of medicines in Norway (FEST) or from internal support registries. These internal registries were based on EMA’s Antimicrobial Sales and Use (ASU) technical implementation protocol

In the next step, data on use and dispensing of antibiotic VMPs and HMPs in 2023 were extracted from VetReg. This included records with the ATCvet and ATC codes (for humans) shown in Supplementary Table 2. These represent the antibiotic VMPs and HMPs that are mandatory to report to EMA [9].

**Fig. 2:**
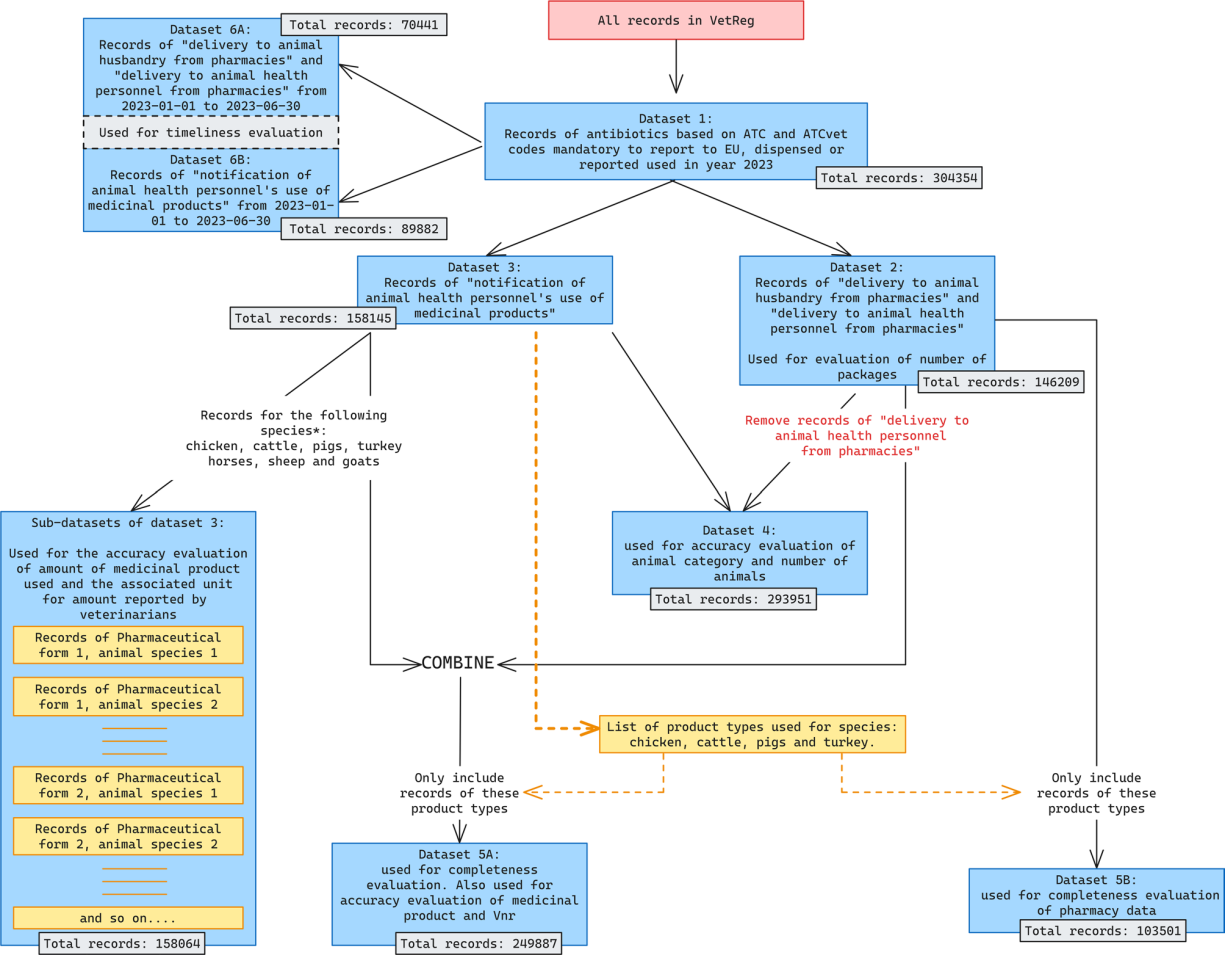
Inclusion and exclusion criteria applied for the eight datasets created for quality analysis. For each dataset the number of records and the purpose of creation of the dataset is given. Sub-datasets from dataset 3 cover each combination of pharmaceutical form and animal species, for the species cattle, pigs, chickens, turkeys, sheep, goats and horses or recorded used for the categories “other poultry” and “other food-producing animals”

Relevant variables for this study were extracted from the use data (Table 1). The final VetReg dataset applied in this study is referred to as Dataset 1 (Fig. 2). The further process of making datasets suitable for various analysis as well as information about the numbers of VetReg-records included in each dataset that have been used for the stepwise approach applied, is presented in in a flow-chart (Fig. 2). Below, the datasets are described further alongside the methodology for the various analysis performed.

The sales dataset used in this study was made by filtering the sales data obtained from NIPH for the ATCvet-codes shown in Supplementary Table 2. Data were supplemented with the same variables from FEST as used for the VetReg-data (Table 1), using the Nordic article number as a common identifier. Furthermore, if more than one Nordic article number were linked to the same product presentation, their sales in terms of number of packages were combined. The term “product presentation” refers to the same medicinal product name, pharmaceutical form, active substance(s), strength and pack size. Further details of sales dataset preparation are given in Supplementary Text 1.

### Quality evaluation

In this study three quality attributes were evaluated: accuracy of the data, completeness of the data, as well as timeliness of the reporting.

### Accuracy

In this context, accuracy means whether data variables reported to VetReg used in this study represent the true values of what was actually dispensed, administered or prescribed to which animal(s), and this term is used synonymously with validity. The occurrence of incomplete records and of inconsistencies regarding which values have been entered for some of the variables are included in the accuracy evaluations.

As a first step, the accuracy of the animal category given was assessed, focusing on categories within the animal species for which data shall be reported in step one of the use data reporting, i.e. cattle, pigs, chickens and turkeys. These categories are presented in Supplementary Table 3. Secondly, the accuracy of the variable “number of animals” was investigated. Knowledge of the validity of this variable was a prerequisite for the downstream calculation methodology used in this study for the evaluation of completeness of use data. Lastly, the accuracy of the variables on the amount of medicinal product reported used and the unit for amount of medicinal product used (relevant for veterinary records), number of packages (relevant for pharmacy records), medicinal product name and Nordic article number were investigated. These variables were chosen as they are important for calculating antibiotic use, in number of packages of the VMP and HMP presentations, before the reporting to EMA.

### Animal category

To validate the data about the animal category reported (for cattle, pigs, chickens and turkeys), data in Fig. 1, A, B and C were used (Fig. 2, Dataset 4). The records “dispensing from pharmacies to veterinarians” (Fig. 1, D) were not included because pharmacies do not receive information about which species the veterinarian will use the medicines for.

The data for the variable animal category were evaluated by looking for missing fields and data that could not be categorised according to the legal requirements for data reporting for 2023 and onwards (Supplementary Table 3). Supplementary Table 4 displays the animal categories for which use was reported to VetReg.

### Number of animals

Data in Fig. 1, A, B and C were used (Dataset 4 in Fig. 2) to evaluate the information given for the number of animals. The records “dispensing from pharmacies to veterinarians” (Fig. 1, D) were not included because pharmacies do not know the number of animals the veterinarian will use the medicines for. The variable was investigated for missing values; the detailed methodology followed is given in Supplementary Text 1.

### Amount of medicinal product reported used and unit for amount of medicinal product reported used

For the accuracy evaluation of the information given for the amount of antibiotic medicinal product reported used, only records reported by veterinarians were included (Fig. 1, A, B) as pharmacies report their sales almost exclusively in number of packages. The evaluation was performed separately for each species and by pharmaceutical form. Figure 2 shows the animal categories for which data were included in the accuracy analysis. In addition to those animal species included in reporting step one (to EMA), records for antibiotic use for horses, sheep and goats were included in the analysis, because this gives information about completeness of the VetReg data in general. All pharmaceutical forms were included in the analysis.

As it was identified that injectables and intramammaries represented the major forms, both in terms of number of records and kilograms of active substance, reported used for the animal species in reporting step one (to EMA), the assessment of the validity of these pharmaceutical forms was more comprehensive. Data cleansing in terms of assumed “wrong” unit for the amount of medicinal product reported used was performed per record prior to evaluation of accuracy of the amount reported used.

To calculate the amount of medicinal product reported used per animal per record, the amount reported per record was divided by the reported number of animals to be treated, as the first step. To evaluate all records for intramammaries, the amount reported was converted into a common unit per record – i.e. number of intramammary applicators per animal. The calculation rules are given in Supplementary Table 5. If the number of applicators reported used was not a whole number, it was assumed that the reported unit for the amount was wrong, and this was changed in the cleansing step as described in Supplementary Fig. 1. To identify likely outliers, the maximum number of intramammaries for treatment per dairy cow was set based on information from the Summary of Product Characteristics (SPC) for each intramammary VMP (presented in Supplementary Table 6). For all other pharmaceutical forms, the use per record was calculated in kilograms of active substance(s) using the calculation rules presented in Supplementary Tables 7A and 7B.

To identify likely errors in the reporting of the amount used to VetReg, a set of validation criteria was made. These varied between species and pharmaceutical forms. For injections, the records for cattle, pigs, sheep and goats were evaluated using information from the SPC for the VMPs in question. The cut-offs for amount of VMPs for cattle, pigs sheep and goats set per Nordic article number are presented in Supplementary Table 8. For records reported for horses, Grubbs test was performed to identify outliers (using p-value of 0.01) [25].

For oral paste, the cut-off value for cattle is provided in Supplementary Text 1. For all other pharmaceutical forms, the cut-off values were set by applying Grubbs test. In cases where there were less than 7 records for a combination of Nordic article number, reporting unit for the amounts and animal species, the Grubbs test could not be performed. These records were manually inspected to identify likely outliers for the amount reported used.

The detailed methodology is given in Supplementary Text 1.

### Number of packages

All entries from pharmacies covered (Dataset 2 in Fig. 2) were checked for the presence of entries that reported dispensing of 0 packages, negative number of packages and decimal separator when part of a package had been dispensed (e.g. 100 mL of a 5 × 100 mL package).

### Medicinal product name and nordic Article number

The accuracy of data in the variables Medicinal product name and Nordic article number were investigated by comparing summarised calculated use (VetReg) with summarised calculated sales (NIPH data), in kilograms per active substance, per product presentation. Thereafter, the use was summarised for each product type (here used as a term for medicinal products with the same medicinal product name, pharmaceutical form and strength(s) of active substance(s)). This was performed on sales and use datasets for product types reported used or dispensed for the animal categories included in EU reporting step 1 (Dataset 5 A in Fig. 2 and Supplementary Table 3). The detailed methodology is given in Supplementary Text 1.

Use of this methodology was only relevant for the product types containing more than one product presentation, for example when one product was available in more than one package size.

### Completeness

The European Centre for Disease Prevention and Control (ECDC) (2014) defines completeness of reporting as the “absence of underreporting”. In this study, it was evaluated to which degree data on all use or dispensing is entered in VetReg, by comparing the use (VetReg) and sales (NIPH data) data for antibiotic VMPs. The occurrence of incomplete records was investigated as part of the accuracy evaluation for the relevant variables.

In order to assess the overall completeness of veterinary use and amounts dispensed by pharmacies to animal owners (Fig. 1, A, B and C), i.e. use data of antibiotic VMPs mandatory to be reported for the animal species in scope for the 2023 EU-call, these were compared to sales data of VMPs for terrestrial animals. Completeness was evaluated per product type, per pharmaceutical form, and in total.

To evaluate the completeness of pharmacy data, sales data was compared with VetReg data from pharmacies (Fig. 1, C and D). The detailed methodology is given in Supplementary Text 1.

### Timeliness

A register’s timeliness is the time between the event and when it is entered in the register. To evaluate if the reporting day impacted the completeness, knowledge about timeliness is important.

From Dataset 1 all records with dates for dispensed or used between 1st January and 30th June were included for the timeliness evaluation performed for each use records reported by veterinarians and each record of medicinal products reported dispensed to animal owners and veterinarians by pharmacies. This created data set 6 A and 6B as shown in Fig. 2, respectively. A limited period for use data was chosen to reduce the bias inflicted by the date on which the data was downloaded from VetReg (15.03.2024). Timeliness was evaluated as the number of days between reported use date and registry date in VetReg.

### Descriptive analysis and statistics

All analysis was performed in R 4.4.0 and RStudio version 2023.06 [26, 27]. The following R packages were used to assist with the analysis: dplyr, janitor, lubridate, stringr, openxlsx, outliers, tidyr and reshape2 [28–34].

Grubbs test was used to identify outliers for the amount of medicinal product used by a veterinarian [25]. This test identifies one outlier at a time, removing it from the dataset and repeating the test until no further outliers are found. However, repeated iterations can alter detection probabilities, and the test is not recommended for sample sizes of six or fewer, as it often labels most points as outliers in smaller datasets.

## Results

### Accuracy

#### Animal category

Of the 293,951 records investigated for accuracy of reported animal category, 304 had no information provided for animal category and all of these originated from pharmacy records. A total of 122,746 of the records were identified as relevant for the 2023-EU data reporting of which 120,094 were from veterinarians and 2,652 from pharmacies. Of these records, it was not possible to assign 5,521 to any of the animal categories given in EU 2021/578. These records did not specify the category for the animal species; for example, records specified “cattle” but did not indicate whether they were categorized as “beef cattle”, “dairy cattle”, or “other cattle”. Of these entries 2,190 were made by pharmacies (82.6% of all pharmacy records relevant for the 2023-EU data reporting) and 3,331 entries by veterinarians (2.7% of all veterinary records relevant for the 2023-EU data reporting). The details are displayed in Table 2. The reason for this was that not all national reporting systems had been updated in accordance with the new requirements [14]. The animal categories for which use data have to be reported are presented in Supplementary Table 4.

**Table 2:** Results of accuracy evaluation of the variable “animal category”, split into veterinary records and records from pharmacies

Results of the evaluation		Number of VetReg records		
Met reporting requirements to VetReg	Reason	Veterinarians’ use	Pharmacies’ dispensing to animal owners	Total number of records
Yes	Animal species should be reported to EMA, and category can be specified	116,763	462	**117**,**225**
	Reported, but animal species not included in first step of reporting to EMA	38,051	132,850	**170**,**901**
No	Animal species should be reported to EMA, but not reported per category	3,331	2,190	**5**,**521**
	No information provided	0	304	304
	**Total number of records**	**158**,**145**	**135**,**806**	**293**,**951**

The variable named animal category in VetReg should be filled with name of species or category within a species. The details required are specified per species. The results are presented as number of records adhering to VetReg’s requirements, subdivided in whether or not the data are relevant for the first reporting step of ASU data to EMA. It also gives number of missing values,

### Number of animals

The number of animals was given for 6% of the records from veterinarians and 98% of the records from pharmacies. A missing entry was assumed to be one animal if individual ID was given, and the number of animals could therefore be established for 68% of all records from veterinarians. The details are presented in Table 3. Of the records where individual ID was given, the number of animals specified were 0 (98,816 records), 1 (52 records), 2 (2 records), 3 (3 records) and 6 (6 records).

**Table 3:** Quality evaluation of the variable “number of animals”

	Number of VetReg records of:		
Results of evaluation	Veterinarians’ use	Pharmacies’ dispensing to animal owners	Total number of records
Number of animals provided	8,911	133,317	**142**,**228**
Number not provided, but animal ID was provided	98,816	0	**98**,**816**
Neither number nor animal ID provided	50,418	2,489	**52**,**907**
**Total number of records**	**158**,**145**	**135**,**806**	**293**,**951**

The evaluation is given as number of records for which number of animals was given, number of animals was not given but animal ID was given and when neither number of animals nor animal ID were given

### Amount of medicinal product used and associated unit for amount of medicinal product reported used by veterinarians

**Fig. 3:**
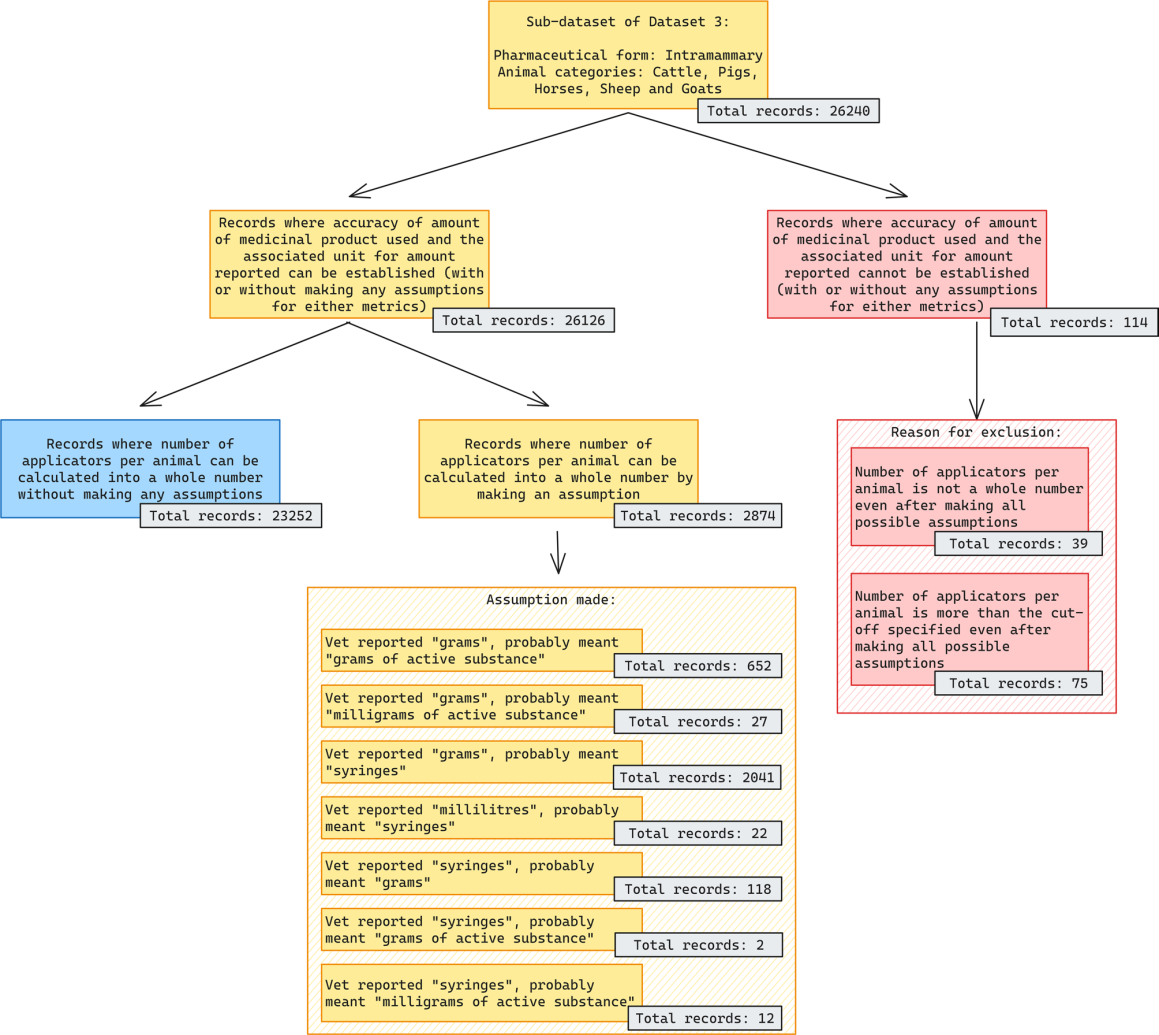
Accuracy evaluation of the variables “reported use” and “unit for of reported use” for intramammaries. The result is presented as the number of records accurately reported, the number of records where use could be established after a data cleansing step and number of records where use was believed to be inaccurately reported. For the two latter groups, results are presented as number of records per type of error corrected and per reason for exclusion, respectively

In total, 26,240 records of intramammaries reported by veterinarians were investigated. By calculating the number of applicators per animal based on the calculation rules set in Supplementary Table 5, it was possible to calculate a whole number of intramammaries for 87% of the records. For 2,874 of the records, changes in the reporting unit for the amounts were made, which then led to use per record being calculated as a whole number of applicators. For 39 records, the number of applicators could not be calculated into whole numbers. For 75 records the calculated number of applicators exceeded the number set as cut-offs. For all these 114 records, the units were assumed to be wrongly recorded. The details are shown as a flowchart in Fig. 3.

For injections, 99.8% of the records were evaluated to be correct without any changes (129,115 of 129,347 entries). For 3 records reported in “piece” (in Norwegian “stk”), the unit reported was assumed to be wrong and changed to “mL” after manual inspection. For 4 records it was not possible to calculate the use, because the unit “dose” was used for reporting the amount, and the size of the dose was then unknown. For 225 records, the reported amount of medicinal product used per animal was above the cut-off values or was flagged in the Grubbs test and therefore assumed to be incorrect after manual inspection. Details can be seen in Table 4.

**Table 4:** Accuracy evaluation of the variables “reported use” and “unit for of reported use” for injections

Results of evaluation	Number of records
Correct unit and number of animals is given	128,907
Correct unit, but number of animals is not given	208
Changed unit from “stk” to “mL”	3
Excluded because unit was “dose”	4
Excluded because quantity exceeded cut-offs	147
Excluded as outliers in Grubbs test (horses)	78
**Total numbers of records**	**129**,**347**

The table provides number of records where accurate unit were given, subdivided into records where number of animals were given or not, the number of records that went through a data cleansing step and the number of records assumed to be inaccurate, including the reason for this assumption

For oral paste, the calculated amount of medicinal product used per animal was evaluated to be correctly given for 96% of the records (585 of 608 records). For five records, the unit for the amounts was assumed to be wrong and changed (veterinarians reported in “kg”, probably meant to report in “g”). For 14 records, use was not possible to calculate, because the unit “dose” (12 records) and “ml” (2 records) were used for reporting. For 4 records, the calculated amount of medicinal product per animal was above the cut-off, identified by Grubbs test and following manual inspection the amount was assumed to be wrong.

For the other pharmaceutical forms, the calculated amount of medicinal product used per animal was evaluated to be correctly given for 98% of the records (1908 of 1948 entries). For 3 entries, the unit reported was believed to be wrong and changed (veterinarians reported “kg”, probably meaning “g”). These three entries were for the pharmaceutical form oral powder for solution. For 15 entries, it was not possible to calculate the use, because the unit “dose” (3 records) and “ml” (12 records) was used for reporting the amount. For 17 records, the calculated amount of medicinal product per animal was above the cut-off, identified by Grubbs test and following manual inspection, the amount was assumed to be wrong. Of the 126 records that could not be evaluated by Grubbs test, 3 were excluded after manual inspection.

If use was given as “dose” for any pharmaceutical form other than intramammaries, the use could not be calculated. There were 19 such records. Further, for some pharmaceutical forms, such as powder for injections and oral paste, if the use was reported in “ml”, the use could not be calculated as well because the total volume of the solution could not be calculated as the SPCs of these medicinal products did not provide information about package size in “ml”. There were 14 such records.

### Number of packages reported dispensed by pharmacies

The number of packages dispensed by pharmacies varied between − 11 and 40 per record. Of 146,209 records in the dataset, there were 1223 entries of zero packages and 66 entries of negative number of packages. All entries with zero and negative number of packages were entries from “Delivery to animal health personnel from pharmacies”. NFSA informed us that they learned from a dialogue with the pharmacies that zero packages meant ordered by veterinarians but not delivered by pharmacies. They had no explanation for the negative numbers. Of the remaining entries where number of packages was more than zero, 2.7% of the entries were of decimal-numbers, thereby revealing that dispensing of partial packages can be reported by pharmacies and received by VetReg.

Pharmacies report data almost exclusively as number of packages; however, 86 records were reported in other units. These were calculated into number of packages using the same calculation rules as for records from veterinarians.

### Medicinal product name and nordic Article number

Ten medicinal product types (medicinal products with the same medicinal product name, pharmaceutical form and strength(s) of active substance(s)) had more than one product presentation (23 different product presentations). Table 5 displays the results of the accuracy analysis, i.e. calculated use was divided by calculated sales in percent per product presentation and per product type. The use compared to sales varied between the various product presentations within a product type, e.g. for one VMP sold in three different package sizes, use was reported for 4%, 16% and 5632% of what was reported sold. When we evaluated use for all package sizes together, use represented 90% of sales data.

**Table 5:** Results of the accuracy evaluation of the variables medicinal product name and nordic Article number

Pharmaceutical form	Medicinal product	Package Size	Nordic article number(s) per product presentation	Use/ sales per product presentation	Use/ sales per product type
Injection	Borgal vet	500 mL	308,144 and 502,070	71.1%	71.4%
Injection	Borgal vet	100 mL	573,998	Not in sales	
Injection	Penovet vet	2500 mL	144,907	3.7%	90.2%
Injection	Penovet vet	100 mL	454,918	5632.0%	
Injection	Penovet vet	500 mL	511,675	16.1%	
Injection	Primopen vet	1000 mL	560,289	60.1%	33.9%
Injection	Primopen vet	1500 mL	529,597	Not in VetReg	
Injection	Streptocillin vet	500 mL	402,735	55.0%	55.0%
Injection	Streptocillin vet	100 mL	145,003	Not in sales	
Injection	Streptocillin vet	2500 mL	402,743	Not in sales	
Injection	Zactran	100 mL	159,386	83.7%	81.6%
Injection	Zactran	50 mL	376,742	16.7%	
Intramammary	Carepen vet	5 applicators	001994 and 482,630	3029.6%	74.7%
Intramammary	Carepen vet	100 applicators	019403 and 486,198	4.6%	
Intramammary	Mastipen vet	100 applicators	027429	50.8%	90.1%
Intramammary	Mastipen vet	200 applicators	27,442	0.0%	
Intramammary	Mastipen vet	5 applicators	76,460	4991.6%	
Oral paste	Norodine vet	260 g	408,074	83.7%	83.7%
Oral paste	Norodine vet	225 g	137,448	Not in sales	
Tablet	Baytril	10 tablets	484,788	103.4%	97.2%
Tablet	Baytril	20 tablets	477,405	84.3%	
Tablet	Baytril vet	20 tablets	019775	61.4%	82.8%
Tablet	Baytril vet	10 tablets	496,174	94.9%	

This accuracy evaluation was only performed when more than one package size was used of the same product presentation (common name, active substance(s), strength, pack size and pharmaceutical form presented as use in percent of sales (NIPH data), per product presentation and per product type (common name, strength of active substance(s) and pharmaceutical form)

### Completeness

The overall completeness in terms of amounts of antibiotics reported to VetReg (use reported by veterinarians and dispensed amounts from pharmacies) compared to sales (reported to NIPH) was calculated to be 85%. Only minor differences were observed in completeness between the most frequently used pharmaceutical forms; 86% for injections, 78% for intramammaries, 84% for oral paste and 91% for tablets. Results per pharmaceutical form are shown in Table 6, while detailed results are shown in Supplementary Table 9.

**Table 6:** Results of the completeness evaluation per pharmaceutical form and in total of 2023 data in VetReg

Pharmaceutical form	Completeness comparing data reported by veterinarians and data reported dispensed from pharmacies to owners to sales data		Completeness comparing data reported dispensed from pharmacies to owners and animal health personnels to sales data	
	Number of records	Completeness (in percent)	Number of records	Completeness (in percent)
Injection	130,401	86.2	8421	56.8
Injection powder	12	Not present in sales data	0	Not present in sales or pharmacy data
Intramammary	26,912	78.3	2467	64.9
Oral solution	141	93.1	115	90.8
Powder for oral solution	85	49.6	18	14.5
Oral paste	6301	83.7	6019	84.1
Oral powder	76	79.6	56	88.6
Tablet	85,797	91.2	86,396	92.2
Intrauterine	162	782.9	9	66.7
**Total**	**249,887**	**85.0**	**103,501**	**63.6**

The results are presented in percent of use compared to sales (NIPH data), subdivided into data to be reported to EU (use reported by veterinarians and pharmacies dispensing of prescriptions to animal owners) and pharmacy reported records in total (pharmacies dispensing to both animal owners and veterinarians)

When the aggregated amounts, dispensed use to animal owners and veterinarians, reported by pharmacies (Fig. 1, C and D) were compared to sales data from NIPH, 64% completeness was observed. This number deviated more by pharmaceutical form compared to the overall completeness: 57% for injections, 65% for intramammaries, 84% for oral paste and 92% for tablets. Results per pharmaceutical form are shown in Table 6, while detailed results are shown in Supplementary Table 10.

Of note is the overall completeness of only 50% for powder for oral solution, which is only used for poultry. The completeness of pharmacy records for this pharmaceutical form was 15%.

### Timeliness

**Fig. 4:**
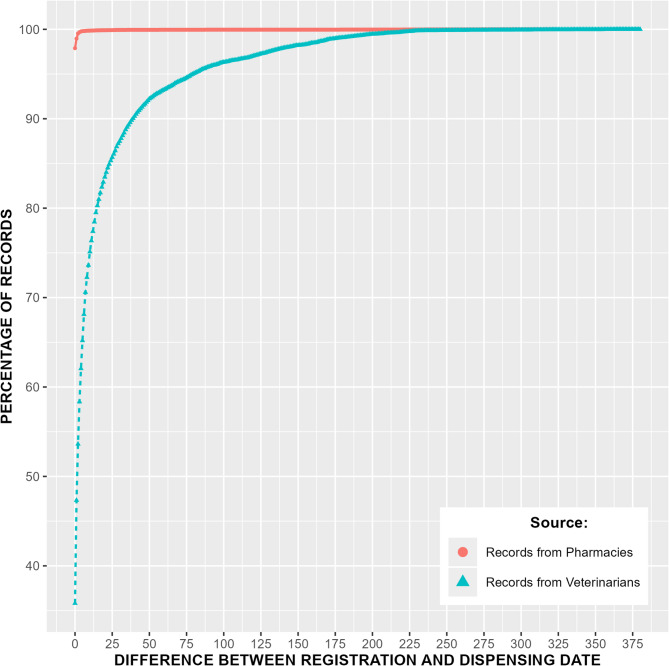
Timeliness of reports to VetReg for antibiotic data. The percentage of records was calculated by dividing the number of records with a difference between registration and dispensing date less than or equal to days on y-axis by the total number of records registered

The results of the timeliness evaluation showed that 70.6% of the veterinary records were reported within the deadline of seven days. For pharmacy records, 99.9% of the records were reported within seven days and 99.6% records were reported within two days. Details of the timeliness evaluation are displayed in Fig. 4.

## Discussion

VetReg is a system suited to receive all data required to fulfill Norway’s obligations for ASU-reporting according to the first two steps of EU regulation 2019/6. Both veterinarians’ and pharmacies’ electronic systems communicate with VetReg and there is a solution available for those not using such systems. For the first year of ASU reporting, the use data were found to be 85% complete. The quality evaluation performed in the current study however shows that VetReg data for 2023 does not provide fully “accurate, complete and consistent” data, as required in the EU Delegated regulation 2021/578 [8, 9]. In order to comply with this in future years, the quality needs to be improved.

The accuracy issues identified for animal category affected the adherence to the regulations [9]. Data that were not reported to VetReg per category, could not be reported to EMA according to regulation [9]. The origin of this error could be either the veterinarians’ prescriptions or the pharmacy’s entry of their dispensing. Missing value for species meant that data could not be used for reporting.

The unit for reporting of amount is not fixed for any medicinal product or product form which led to an inconsistent choice of units for reporting use of the same product. Moreover, records with presumably wrong units were identified; for example, “kilogram” when it presumably should have been “gram”. In such cases, the difference in use will differ by a magnitude of a thousand if left uncorrected. The data cleansing, however, may create new errors if issues were incorrectly addressed. Use of various units for reporting were recently identified to lead to a quality issue with fish vaccine data in VetReg as well [19].

Wrong units given for the amounts were assessed to be the most common error for intramammaries (11% of intramammary records), including the reporting of gram active substance instead of gram product (2.5% of intramammary records). This was assumed to partly be due to the strength of active substance being shown as part of the name when choosing medicinal product from a drop-down menu in the veterinary practice management software programs. This error can only be identified for intramammaries containing only one active substance, as products containing multiple active substances contain different strengths of each substance. The unit issues in VetReg have also been described in previous quality evaluations, including one focusing on quality of data for intramammaries [16–18]. Previous evaluations concluded that, when compared to the sales data for these forms, the summarized use of antibiotics calculated from records of reported use of intramammaries or oral paste showed a huge difference. Sanders et al. (2020) claimed that limiting the options per input field would improve the data quality [14]. An application of this can be in the technical solutions to limit the possible reporting unit to one per pharmaceutical form, for example to “applicators” for intramammaries.

Another accuracy issue was the reporting of presumably wrong amounts. This can both underestimate and overestimate the use compared to actual use. Corrections of amounts by exclusion of data will decrease the calculated use compared to actual use. A conservative approach was therefore chosen for the exclusion of data during the downstream analysis; i.e., the cut-offs were set at a very high use. The Danish use data collection system VetStat has a system where all reporting veterinarians get reports of their own use data and must correct records if errors are found [35]. Most likely this quality check reduces the number of accuracy issues of e.g. amounts used that are left uncorrected in the register. A change in regulation as well as technical solutions are needed for this to become mandatory in Norway.

In a previous study it was shown that when pharmacies reported dispensed amounts as part of packages with decimal numbers, the amounts appeared in VetReg as rounded whole numbers [16]. Errors in the system were corrected, and this was no longer an issue for 2023 data. This shows how quality improvements can be made following quality evaluations.

The accuracy of the medicinal product name was found to be better than for the Nordic article number. This observation agreed with the findings in fish vaccine quality assessment of VetReg, where combining vaccines available in various pack sizes improved the overall coverage of the vaccines compared to evaluating per individual product presentation [19]. The Nordic article number was indeed for the medicinal products reported used, but the choice of package presentation, and hence Nordic article number, seemed to be partly random. Use will therefore be reported to EMA of other package presentations than those actually used, if use is reported as found in VetReg. The calculated use, in kilograms of active substance, will however not be affected, since the product name, active substance(s), strength and pharmaceutical form are the same and veterinarians report their use in units like gram and milliliters and not in in number of packages.

The missing information on the number of animals did not directly affect the calculation of the amount used, but added insecurity to the accuracy evaluations of the amounts used. Missing number of animals is an example where technical solutions possibly could improve the data quality. This could also be relevant for several of the other issues identified in the current study, such as unit for amount.

The quantities of antibiotic VMPs found in the VetReg-data investigated in the current study did not fully match sales data of the same VMPs. The main reason for the use data not being fully complete is assumed to be due to underreporting. Additionally, one of the minor reasons for data not being complete is the inbuilt lack of completeness since it is not mandatory for veterinarians to report use in companion animals. Lack of these data was also identified as a quality issue in the Danish VetStat [35]. Another minor reason for reduced completeness is the exclusion of data after the accuracy evaluation; this excluded 0.3% (*N* = 397) of the veterinary records. A third minor reason is that, due to capacity constraints, only seven of the 23 terrestrial animal species were included in the analysis of veterinary records, excluding 0.06% (*N* = 88) of the data.

Waste of unused medicine and products bought but not used in the same year may also have some impact. It is, however, assumed that the amount of stocking of products is evened out across years. The lower completeness found for pharmacy records was somewhat surprising, since reporting to VetReg is a part of the book-keeping system for most pharmacies. Further investigations are needed to identify why pharmacies are underreporting the use data for animals.

Of special note is the low (50%) overall completeness of the pharmaceutical form oral powder for solution. The completeness of only pharmacies dispensing to animal owners or veterinarians, compared to sales data, was even lower (15%). This affects the completeness of use data for chickens and turkeys, which are the two species this pharmaceutical form is used for. The reason could be underreporting by pharmacies and possibly also veterinarians.

The investigation of timeliness of VetReg showed that 29% of all veterinary records were reported after the deadline of seven days set by NFSA. Since it took approximately 225 days before all veterinary records for 2023 were reported, extracting the data on March 15 resulted in timeliness issues that affected data completeness. Pharmacies report the data to VetReg faster than veterinarians, probably because the data recording is a part of their dispensing process, while veterinarians manually fill in information about their use of medicines in their practice management software programs, possibly some time after the treatment occurred.

With regard to investigating consistency, we only had access to the data in the files shared by NFSA. It could therefore not be investigated if data changed through the data transfer stages.

EMA’s first ASU report revealed that quality issues with completeness of AMU data are shared with many other European countries [11]. Varying coverage, leading to use data not representing actual use, was given as the reason to why use data were not presented quantitatively in the report. The type of quality evaluation performed in the current study might therefore be useful also in other countries to gain deeper insight that could aid the quality improvement work. The AMU data collection systems are however set up differently in other countries [11] and hence quality evaluations must be performed tailored to the relevant system and to the desired output of the evaluation.

## Conclusions

Antibiotic use data for animals in Norway in 2023 could be reported to EMA using VetReg data and for the first year of reporting use data were found to be 85% complete. The data were, however, not fully accurate and complete. This study revealed several specific accuracy issues and issues with timeliness of reporting. These findings provide the basis for targeted quality improvement measures and can be transformed to metrics suitable to track the progress of the ongoing quality improvement work. All issues identified have been reported to the data owner to assist in their efforts towards improved data quality.

## Electronic supplementary material

Below is the link to the electronic supplementary material.

**Supplementary Material 1:** Supplementary Figure 1

**Supplementary Material 2:** Supplementary Table 1

**Supplementary Material 3:** Supplementary Table 2

**Supplementary Material 4:** Supplementary Table 3

**Supplementary Material 5:** Supplementary Table 4

**Supplementary Material 6:** Supplementary Table 5

**Supplementary Material 7:** Supplementary Table 6

**Supplementary Material 8:** Supplementary Table 7A

**Supplementary Material 9:** Supplementary Table 7B

**Supplementary Material 10:** Supplementary Table 8

**Supplementary Material 11:** Supplementary Table 9

**Supplementary Material 12:** Supplementary Table 10

**Supplementary Material 13:** Supplementary Text 1

## Data Availability

The data used in this study, VetReg and wholesaler data, are not publicly available. They were made available for NVI via an agreement with NFSA (VetReg) and NIPH (wholesaler data). The data can also be requested by others from the same data providers.

## Abbreviations

AMU: Antimicrobial use
ASU: Antibiotic Sales and Use
ATC: Anatomical Therapeutic Chemical
ATCvet: Anatomical Therapeutic Chemical for veterinary medicine
ECDC: European Centre for Disease Prevention and Control
EEA: European Economic Area
EMA: European Medicines Agency
ESVAC: European Surveillance of Veterinary Antimicrobial Consumption
EU: European Union
FEST: Forskrivnings- og ekspedisjonsstøtte (support registry for prescribing and dispensing)
HMP: Human Medicinal Products
NIPH: Norwegian Institute of Public Health
NVI: Norwegian Veterinary Institute
NFSA: Norwegian Food Safety Authority
NoMA: Norwegian Medicinal Products Agency
SPC: Summary of Product Characteristics
VetReg: Norwegian Veterinary Prescription Register
VMP: Veterinary Medicinal Products
WOAH: World Organization for Animal Health
