# Supplementary material for: Quality assessment of antibiotic use data in the Norwegian veterinary prescription register for 2023
Source: BMC Vet Res. 2025 Aug 7;21:510. doi: 10.1186/s12917-025-04903-9 (PMC12333288; doi:10.1186/s12917-025-04903-9)
Supplement: Supplementary file 13 — Supplementary Material 13: Supplementary Text 1 [file 12917_2025_4903_MOESM13_ESM.docx]

**Supplementary Text 1**

**This is a more comprehensive version of the methods section. Text included in the main text is also included in the supplementary text, to provide context to the additional text.**

**References to figures, tables and references are identical to those given in the main text.**

**Methods**

**Data sources**

Data on the use of medicinal products in animals has been collected by NFSA through VetReg and the type of data reported to VetReg are shown in Figure 1. It is mandatory for pharmacies to report all medicines dispensed to animal owners (i.e. for all animal species) and to veterinarians for use in their practice. Veterinarians must report all their use and administration of medicines to food-producing animals (including horses), while it is voluntary to report use for other species kept or bred e.g. companion animals and fur animals. Veterinarians are allowed to use or prescribe medicines for other species and other indications than these are authorised for, including HMPs, however, such use has to be in accordance with Article 112-114 of EU regulation 2019/6 (8).

Data to VetReg are submitted via pharmacies’ accounting systems and veterinarians’ practice management software programs, respectively, and via system-to-system communication with VetReg. The data can also be reported to VetReg using NFSA’s web portal. NFSA accepts corrections in VetReg within six months of the original reporting.

In the following, one line in VetReg is referred to as one record; this applies both for records from pharmacies and from veterinarians. One record contains information about the use or dispensing of one VMP or HMP on one occasion to one animal or a group of animals. Records representing veterinarians’ use and pharmacies dispensing to animal owners, respectively, should provide the species of the treated animal, and since 2023 additionally provide the animal category for cattle, pigs, chickens and turkeys, e.g. differentiate between dairy cattle and beef cattle. All variables in a record are shown in Supplementary Table 1.

For pharmacies, the system is designed for reporting the amount of the VMP and HMP dispensed to the animal owners and veterinarians, almost exclusively as number of packages (and part of packages when relevant). The VetReg variable name is “number_of_packages” (Table 1). Veterinarians report the amount in e.g. mL, g or dose of the VMP or HMP. The VetReg variables are “dispensed_quantity” and “dispensed_unit” (Table 1).

For veterinarians, the use must be reported to VetReg within seven days, while no reporting deadline has been established for prescriptions dispensed by pharmacies, to the best of our knowledge (21). The reason behind this specific discrimination between veterinarians and pharmacies is not known to us.

Access to VetReg is restricted but is made available for the Norwegian Veterinary institute (NVI) by NFSA through a mutual agreement. The first VetReg data for 2023 were made available for NVI on 11.01.2024 and VetReg-data for NVI was thereafter updated daily. For this study, the data were downloaded as csv-files on 15.03.2024, meaning that 2023-data reported to VetReg later than 15.03.2024 were not included in the current study.

The Norwegian Medicinal Products Agency (NoMA) owns and maintains a database, called FEST, containing information about medicinal products. The main purpose of this database is to support prescribing and dispensing of medicines in Norway (in Norwegian: Forskrivnings- og ekspedisjonsstøtte, abbreviated FEST) (22). The FEST database was used to supplement VetReg data and sales data as referred to in the section “Preparation of data for further analysis” below.

To assess the accuracy and completeness of antibiotic VMP use data reported to VetReg, covering the Anatomical Therapeutic Chemical veterinary (ATCvet) codes for which use is mandatory to be reported to the European Medicines Agency according to the delegating regulation (EU 2021/578) (see Supplementary Table 2), sales data for antibiotic VMPs with the same codes were used. These data were collected from the Norwegian Institute of Public Health (NIPH) as wholesalers are mandated to report their sales of VMPs (and HMPs) to pharmacies directly to NIPH. These data are used for the reporting of sales data to EMA. Sales of HMPs by wholesalers were excluded from this analysis because it is not known if the HMPs sold to pharmacies are used for humans or animals. The variables collected on sales of antibiotic VMPs are, among others, ATCvet code, name of the VMP and the number of packages sold per Nordic article number (common article numbers per VMP and HMP used by all stakeholders) (23).

For the wholesalers, the deadline for the reporting of annual sales data for the preceding year to NIPH is January 15^th^. NIPH provided NVI with sales data for 2023 on 06.02.2024 for the ATCvet codes shown in Supplementary Table 2, which covers data of antibiotic VMPs mandatory to be reported to EMA (9)***.***

**Preparation of data for further analysis**

As a first step, VetReg data were supplemented with FEST data using the Nordic article number as the common identifier. Variables added from FEST are shown in Table 1. Each pharmaceutical form provided in FEST was thereafter assigned to the same groups of pharmaceutical forms as those given in the Antimicrobial Sales and Use (ASU) technical implementation protocol (24). This grouping decreased the number of pharmaceutical forms.

In the next step, data on use and dispensing of antibiotic VMPs and HMPs in 2023 were extracted from VetReg. This included records with the ATCvet and ATC codes (for humans) shown in Supplementary Table 2. These represent the antibiotic VMPs and HMPs that are mandatory to report to EMA (9).

Relevant variables for this study were extracted from the use data (Table 1). The final VetReg dataset applied in this study is referred to as Dataset 1 (Figure 2). The further process of making datasets suitable for various analysis as well as information about the numbers of VetReg-records included in each dataset that have been used for the stepwise approach applied, is presented in in a flow-chart (Figure 2). Below, the datasets are described further alongside the methodology for the various analysis performed.

The sales dataset used in this study was made by filtering the sales data obtained from NIPH for the ATCvet-codes shown in Supplementary Table 2. Data were supplemented with the same variables from FEST as used for the VetReg-data (Table 1), using the Nordic article number as a common identifier. Furthermore, if more than one Nordic article number were linked to the same product presentation, their sales in terms of number of packages were combined. The term “product presentation” refers to the same medicinal product name, pharmaceutical form, active substance(s), strength and pack size. Of note is that in four cases, a product presentation was associated with a national article number (local article number not included in FEST) instead of the Nordic article number (23). This might be the case if the VMP or HMP do not have marked authorization in Norway, but are imported on special license provided by NoMA. It is however also possible to obtain a Nordic article number also for these products. After NIPH’s communication with wholesalers, the Nordic article number for three of the National article numbers were identified and assigned. Only one package had been sold of the medicinal product still missing Nordic article number and this record was excluded from the dataset.

**Quality evaluation**

In this study three quality attributes were evaluated: accuracy of the data, completeness of the data, as well as timeliness of the reporting.

**Accuracy**

In this context, accuracy means whether data variables reported to VetReg used in this study represent the true values of what was actually dispensed, administered or prescribed to which animal(s), and this term is used synonymously with validity. The occurrence of incomplete records and of inconsistencies regarding which values have been entered for some of the variables are included in the accuracy evaluations.

As a first step, the accuracy of the animal category given was assessed, focusing on categories within the animal species for which data shall be reported in step one of the use data reporting, i.e. cattle, pigs, chickens and turkeys. These categories are presented in Supplementary Table 3. Secondly, the accuracy of the variable “number of animals” was investigated. Knowledge of the validity of this variable was a prerequisite for the downstream calculation methodology used in this study for the evaluation of completeness of use data. Lastly, the accuracy of the variables on the amount of medicinal product reported used and the unit for amount of medicinal product used (relevant for veterinary records), number of packages (relevant for pharmacy records), medicinal product name and Nordic article number were investigated. These variables were chosen as they are important for calculating antibiotic use, in number of packages of the VMP and HMP presentations, before the reporting to EMA.

**Animal category**

To validate the data about the animal category reported (for cattle, pigs, chickens and turkeys), data in Figure 1, A, B and C were used (Figure 2, Dataset 4). The records “dispensing from pharmacies to veterinarians” (Figure 1, D) were not included because pharmacies do not receive information about which species the veterinarian will use the medicines for.

The data for the variable animal category were evaluated by looking for missing fields and data that could not be categorised according to the legal requirements for data reporting for 2023 and onwards (Supplementary Table 3). Supplementary Table 4 displays the animal categories for which use was reported to VetReg.

**Number of animals**

Data in Figure 1, A, B and C were used (Dataset 4 in Figure 2) to evaluate the information given for the number of animals. The records “dispensing from pharmacies to veterinarians” (Figure 1, D) were not included because pharmacies do not know the number of animals the veterinarian will use the medicines for.

As this information is important to calculate the amount of medicinal product reported used per animal per record, it was investigated if an individual animal ID was given for the animal ID-variable when the number of animals were not given. In such cases, the number of animals is assumed to be one. In addition, it was checked if the numbers of animals specified when an individual animal ID was given.

**Amount of medicinal product reported used and unit for amount of medicinal product reported used**

For the accuracy evaluation of the information given for the amount of antibiotic medicinal product reported used, only records reported by veterinarians were included (Figure 1, A, B) as pharmacies report their sales almost exclusively in number of packages. The evaluation was performed separately for each species and by pharmaceutical form. Figure 2 shows the animal categories for which data were included in the accuracy analysis. In addition to those animal species included in reporting step one (to EMA), records for antibiotic use for horses, sheep and goats were included in the analysis, because this gives information about completeness of the VetReg data in general. All pharmaceutical forms were included in the analysis.

As it was identified that injectables and intramammaries represented the major forms, both in terms of number of records and kilograms of active substance, reported used for the animal species in reporting step one (to EMA), the assessment of the validity of these pharmaceutical forms was more comprehensive. Data cleansing in terms of assumed “wrong” unit for the amount of medicinal product reported used was performed per record prior to evaluation of accuracy of the amount reported used.

To calculate the amount of medicinal product reported used per animal per record, the amount reported per record was divided by the reported number of animals to be treated, as the first step. If the number of animals was not given, but the individual animal ID was provided, it was assumed to be one animal. When information about the individual ID was not provided either, it was assumed that the amount reported was for use in one animal. In these cases, a note with this assumption was added to the data record and these records were subsequently addressed during the final steps of evaluation.

To evaluate all records for intramammaries, the amount reported was converted into a common unit per record – i.e. number of intramammary applicators per animal. This calculation for number of applicators per animal was performed using various calculation rules depending on the combination of unit for amount for reporting (provided by veterinarians) and unit for amount per package (provided by FEST). The calculation rules are given in Supplementary Table 5. The reason for choosing applicators as unit was the assumption that only a whole number of applicators were used. This assumption was made because only use of whole applicators is mentioned in the SPCs of intramammary VMPs. If the number of applicators reported used was not a whole number, it was assumed that the reported unit for the amount was wrong, and this was changed in the cleansing step as described in Supplementary Figure 1.

To identify likely outliers, the maximum number of intramammaries for treatment per dairy cow was set based on information from the Summary of Product Characteristics (SPC) for each intramammary VMP: Number of applicators needed for treatment for twice the longest duration of treatment given in the SPC, assuming treatment of all teats (four for cattle and two for sheep and for goats). Records giving a higher number for the use of intramammary applicators than this maximum number were defined as an outliers and cleansed (all cleansing steps are presented in Supplementary Figure 1). The maximum number of applicators set per Nordic article number for cattle are presented in Supplementary table 6.

For all other pharmaceutical forms, the use per record was calculated in kilograms of active substance(s). Specific calculation rules were made per pharmaceutical form, for each combination of unit used for reporting by veterinarians, unit for amount per package and the unit for the strength of the active substance. Information for the latter two originated from FEST. All calculation rules are presented in Supplementary Tables 7A and 7B.

Generally, the amount reported used per record, in weight of active substance, was calculated by multiplying the reported use with the strength of the active substance(s) for the VMP or HMP in question. For some antibiotics VMPs, the strength is given for a chemical derivative of the active substance – for example procaine benzylpenicillin for which the active substance is benzylpenicillin. To present use data for the active substance, the calculated amounts of the derivatives was multiplied with the corresponding conversion factors presented in Annex 3 of the Antimicrobial Sales and Use (ASU) technical implementation protocol per 29 January 2024.

To identify likely errors in the reporting of the amount used to VetReg, a set of validation criteria was made. These varied between species and pharmaceutical forms. For injections, the records for cattle, pigs, sheep and goats were evaluated using information from the SPC for the VMPs in question. The highest dose per animal assumed to be clinically relevant for the animal species in question was calculated. These doses were calculated by doubling the highest dosing specified in the SPC multiplied with the highest number of treatment days for animals with the highest assumed weights (1300 kg for cattle, 350 kg for pigs, 160 kg for sheep and 130 kg for goats). The assumptions of weights were made consulting animal species experts at NVI. The cut-offs for amount of VMPs for cattle, pigs sheep and goats set per Nordic article number are presented in Supplementary Table 8. All records with reported use for the VMP above this value were defined as outliers. For records reported for horses, Grubbs test was performed to identify outliers (using p-value of 0.01) for each Nordic article number and reporting unit for amount reported used by veterinarians, e.g. for a specific Nordic article number reported in unit “mL” (25). All the records for which the amount reported used was higher than the cut-off value, were defined as outliers and manually inspected before exclusion. For Nordic article number and reporting unit with fewer than seven records, the Grubbs test could not be performed. These records were manually inspected to identify presumably wrongly reported amounts.

For oral paste records for which the amounts were given in the unit “kg”, it was assumed, after inspection of records that the veterinarian had intended to report “gram”. After implementing this assumption, the amount (grams) reported used per cattle (regulation allows for use in other species than horses, given that a set of criteria are met) were evaluated using the same approach to establish a cut-off value, as for injections. This gave a cut-off value of 1,638 grams for the oral paste used (Norodine vet). All reported use per animal higher than this was defined as outliers and manually inspected prior to exclusion. In the case of records reporting use of oral paste for horses, Grubbs test was used to identify outliers, as previously described.

For all other pharmaceutical forms, the cut-off values were set by applying Grubbs test as followed in case of horses for injections and oral paste. This test was applied per combination of animal species, Nordic article number and reported unit for the amounts, e.g. for a specific Nordic article number reported used for chickens, reported in grams. As previously noted, in cases where there were less than 7 records for a combination of Nordic article number, reporting unit for the amounts and animal species, the Grubbs test could not be performed. These records were manually inspected to identify likely outliers for the amount reported used.

**Number of packages**

All entries from pharmacies covered (Dataset 2 in Figure 2) were checked for the presence of entries that reported dispensing of 0 packages, negative number of packages and decimal separator when part of a package had been dispensed (e.g. 100 mL of a 5 x 100 mL package).

**Medicinal product name and Nordic article number**

The accuracy of data in the variables Medicinal product name and Nordic article number were investigated by comparing summarised calculated use (VetReg) with summarised calculated sales (NIPH data), in kilograms per active substance, per product presentation. Thereafter, the use was summarised for each product type (here used as a term for medicinal products with the same medicinal product name, pharmaceutical form and strength(s) of active substance(s)). This was performed on sales and use datasets for product types reported used or dispensed for the animal categories included in EU reporting step 1 (Dataset 5A in Figure 2 and Supplementary table 3).

The amounts reported used (kilograms of active substance) were summarised after data cleansing, i.e. all units for the amount of medicinal product used assumed to be wrong were corrected, as previously described. In addition, if number of animals were known (a number and/or individual ID was given), all identified outliers were excluded as part of the data cleansing.

Use of this methodology was only relevant for the product types containing more than one product presentation, for example when one product was available in more than one package size. The use and sales were calculated in kilograms per active substance per product presentation and thereafter summarized per product type.

All calculation rules are presented in Supplementary Table 5, 7A and 7B.

**Completeness**

The European Centre for Disease Prevention and Control (ECDC) (2014) defines completeness of reporting as the “absence of underreporting”. In this study, it was evaluated to which degree data on all use or dispensing is entered in VetReg, by comparing the use (VetReg) and sales (NIPH data) data for antibiotic VMPs. The occurrence of incomplete records was investigated as part of the accuracy evaluation for the relevant variables.

In order to assess the overall completeness of veterinary use and amounts dispensed by pharmacies to animal owners (Figure 1, A, B and C), i.e. use data of antibiotic VMPs mandatory to be reported for the animal species in scope for the 2023 EU-call, these were compared to sales data of VMPs for terrestrial animals. Completeness was evaluated per product type, per pharmaceutical form, and in total.

The same sales and use data as used for the accuracy evaluation of medicinal product name and Nordic article number were used. This includes applying the same cleansing step. The only exception was that the completeness assessment also included product types containing only one product presentation, i.e. VMPs available in only one package size. Use and sales, in kilograms, were calculated per active substance per product presentation by applying the calculation rules shown in Supplementary Tables 5, 7A and 7B. Completeness was not evaluated per product presentation because of accuracy issues at that level.

To evaluate the completeness of pharmacy data, sales data was compared with VetReg data from pharmacies (Figure 1, C and D) for the same product types as in the completeness evaluation described above.

**Timeliness**

A register's timeliness is the time between the event and when it is entered in the register. To evaluate if the reporting day impacted the completeness, knowledge about timeliness is important.

From Dataset 1 all records with dates for dispensed or used between 1^st^ January and 30^th^ June were included for the timeliness evaluation performed for each use records reported by veterinarians and each record of medicinal products reported dispensed to animal owners and veterinarians by pharmacies. This created data set 6A and 6B as shown in Figure 2, respectively. A limited period for use data was chosen to reduce the bias inflicted by the date on which the data was downloaded from VetReg (15.03.2024). Timeliness was evaluated as the number of days between reported use date and registry date in VetReg.

**Descriptive analysis and statistics**

All analysis was performed in R 4.4.0 and RStudio version 2023.06 (26, 27). The following R packages were used to assist with the analysis: dplyr, janitor, lubridate, stringr, openxlsx, outliers, tidyr and reshape2 (28–34).

Grubbs test was used to identify outliers for the amount of medicinal product used by a veterinarian (25). This test identifies one outlier at a time, removing it from the dataset and repeating the test until no further outliers are found. However, repeated iterations can alter detection probabilities, and the test is not recommended for sample sizes of six or fewer, as it often labels most points as outliers in smaller datasets.
